# Perceived physical activity barriers related to body weight status and sociodemographic factors among Malaysian men in Klang Valley

**DOI:** 10.1186/1471-2458-13-275

**Published:** 2013-03-26

**Authors:** Suraya Ibrahim, Norimah A Karim, Ng Lai Oon, Wan Zurinah Wan Ngah

**Affiliations:** 1 Nutrition Programme, Faculty of Health Sciences, Universiti Kebangsaan Malaysia, Kuala Lumpur, Malaysia; 2 Health Psychology Programme, Faculty of Health Sciences, Universiti Kebangsaan Malaysia, Kuala Lumpur, Malaysia; 3 Department of Biochemistry, Faculty of Medicine, Universiti Kebangsaan Malaysia, Kuala Lumpur, Malaysia; 4 School of Healthcare Sciences, Faculty of Health Sciences, Universiti Kebangsaan Malaysia, Jalan Raja Muda Abdul Aziz, 50300 Kuala Lumpur, Malaysia

**Keywords:** Physical activity, Barriers, Malaysian, Men, BMI, Waist circumference

## Abstract

**Background:**

Physical inactivity has been acknowledged as a public health issue and has received increasing attention in recent years. This cross-sectional study was conducted to determine the barriers to physical activity among Malaysian men. These barriers were analyzed with regards to sociodemographic factors, physical activity level, BMI and waist circumference.

**Methods:**

Subjects in this study included 308 Malay men and 422 Chinese men aged 20 years and older. Subjects completed the International Physical Activity Questionnaire (IPAQ) and a questionnaire on barriers to physical activity, categorized into personal and psychological, physical and social environment barriers. Weight, height and waist circumference were also measured and BMI was calculated.

**Results:**

Descriptive analyses showed that 79.3% of subjects were married, 52.1% had secondary educational level, 68.8% were still working, and 39.7% had household income between RM1500 to RM3500. The perception that other recreational activities with family and friends were more fun was the most frequently reported barrier, followed by weather, lack of discipline, lack of free time, lack of money, and lack of friends. Marriage status, educational level, household income, BMI, and physical activity status were shown to be associated with perceived barriers.

**Conclusions:**

To increase participation in physical activity, policy makers should consider significant personal, social and environmental barriers when developing appropriate intervention programmes. Health-promoting strategies that increase awareness, knowledge, skills and motivation related to physical activity are required.

## Background

Due to the increasing prevalence of physical inactivity especially among adults, strategies intended to increase physical activity levels have been promoted in many countries [1,2]. In Malaysia, the Ministry of Health had started the Healthy Lifestyle Campaign in 1991, whereby it has been organized with different themes throughout the years, including the Exercise and Fitness Promotion in 1998 [3]. In addition, in the recent Malaysian Dietary Guidelines [4], individuals are encouraged to perform at least 30 minutes of moderate intensity physical activity at least five days a week, although daily physical activity is preferable.

Majority of Malaysians do not meet this recommendation for physical activity. Most Malaysians are not active, and only a small percentage participates in regular and adequate physical activity [5], which may be due to the rapid industrialisation and urbanisation in Malaysia for the past several decades [6]. The prevalence of physical inactivity among Malaysian men is 37% [6]. This shows that being physically active could be a barrier in these Malaysian men. Intra-personal, social and physical environmental factors can affect physical activity behaviours, and these determinants differ across the life course [7]. Therefore, it is important to identify and understand barriers to physical activity in an effort to plan effective interventions.

Studies on physical activity barriers have been conducted in several countries, including the United States, Australia, Japan and Brazil [8-11]. In a review of the correlates of adults’ participation in physical activity, perceived environmental or social barriers were inversely associated with physical activity level [12]. However, the barriers and the extent of their association with physical inactivity depend on the population studied [11]. For example, the Brazilian study found that lack of money and feeling too tired were the most frequently reported barriers to physical activity [11]. While in Japan, the most significant perceived barrier was lack of time [9].

In Malaysia, studies on physical activity barriers among adults are still lacking. A study on the association between socio-demographic and psychosocial factors with physical activity in Petaling Jaya, Selangor among working women has found that physical activity barriers were associated with physical activity level [13]. However this research did not discuss further about the types of barriers that have influenced physical activity. Furthermore, perceived barriers among men may be different from women. Therefore, due to lack of published information on the barriers to physical activity particularly among men, the aim of this study is to identify perceived physical activity barriers and their association with socio-demographic factors, BMI, waist circumference and physical activity status among men aged 20 years and older.

## Methods

### Study design

This cross-sectional study was conducted as part of the Malaysian Aging Males Study [14] which aimed at assessing the nutritional, oxidative, cardiovascular, bone health, and physical activity status of Malaysian men in the central region of Peninsular Malaysia. Purposive sampling technique was adopted, in which the selection of subjects was based on predetermined selection criteria and the willingness or capacity of subjects to participate. Subjects’ recruitment was conducted from September 2009 to September 2011 using public announcements via major newspapers, radio broadcasts, community centers, mosques and flyers. Specific inclusion and exclusion criteria were attached in the advertisements. Prior to the screening, interested subjects would be contacted to ensure they fulfilled the listed criteria and only qualified subjects were allowed to attend the screening session. During the screening session, qualified physicians performed the physical examination and examined the medical history of the subjects to ensure they fulfilled all criteria listed. Sample size calculation showed that a minimum number of 738 subjects were required. This study was approved by the Ethics Committee of Universiti Kebangsaan Malaysia Medical Centre.

### Subjects

All eligible subjects for the Malaysian Aging Males Study were included in this study. A total of 398 Malay men and 442 Chinese men (a total of 840 subjects) aged 20 years and older residing in the central region of Peninsular Malaysia (Kuala Lumpur, Petaling Jaya, Shah Alam, and Klang) were recruited. The exclusion criteria of the subjects were (1) subjects who presented with any apparent signs of mobility impairment like needing walking assistance; (2) subjects who did not complete the anthropometric measurements; (3) subjects who were unable to complete the demographic, International Physical Activity Questionnaire (IPAQ) and physical activity barriers questionnaire. All subjects were screened using a detailed demographic questionnaire adopted (with permission) from the Malaysian Cohort Study. Written informed consent was obtained after information pertaining to this research was explained to the subjects.

Subjects were classified into their respective age clusters (young, middle age, and elderly) according to convention. The elderly population was defined using the World Health Organization standard for developing countries, as population that had reached retirement age [15], which was around 60 years in Malaysia. Subjects aged 40–59 years were classified as “middle-age,” and this cutoff was supported by other researchers studying middle-aged population in Malaysia [16,17]. This was also supported by Rampal et al. [18], who stated that a transition of lifestyle (from less sedentary to sedentary) and body anthropometry was seen in Malaysian men aged 40–59 years, and this could potentially affect their health status [18]. The remaining subjects aged 20–39 years were categorized as young adults.

### Socio-demographics and anthropometry

A detailed demographic questionnaire adopted with permission from Malaysian Cohort study was used to obtain socio-demographic information, including age, ethnicity, marital status, educational level, occupation and monthly household income. Anthropometric measurements included height, body weight and waist circumference. The standing height of the subjects without shoes was measured using a portable stadiometer and was recorded to the nearest 0.1 cm. The weight of the subjects with light clothing but without shoes was determined using a standardized balanced beam scale and was recorded to the nearest 0.1 kg. The body mass index (BMI) was calculated using the formula: BMI (kg/m^2^) = body weight (kg) / (body height × body height) (m^2^). A soft measuring tape was used to measure waist circumference to the nearest 0.1 cm. The waist circumference was measured between the lowest rib margin and the iliac crest while subjects maintained a standing position. Subjects were classified based on their BMI and waist circumference value. Generally, subjects with BMI <18.5 kg/m^2^ were underweight, 18.5–24.9 kg/m^2^ were normal, 25.0–29.9 kg/m^2^ were overweight, and ≥30.0 kg/m^2^ were obese [19]. The waist circumference cutoff point for abdominal obesity in Malaysian men was 90 cm, and it had been used by researchers in this country [20].

### Physical activity assessment

Physical activity level of the subjects was evaluated using the International Physical Activity Questionnaire (IPAQ short form). This questionnaire was publicly available, and no permission was required to use it [21]. All subjects were required to answer the questionnaire consisting of four parts, which included the number of days per week and minutes per day spent on (1) vigorous intensity activity, (2) moderate intensity activity, (3) walking for at least 10 min at one time, and (4) hours spent sitting and/or lying down (excluding sleeping) per day. The physical activity score was calculated as the weekly time spent (in minutes) in moderate activities (including walking) plus twice the weekly time spent in vigorous activities [22]. Individuals with a score of 0 were considered sedentary; those with scores of 10 to 149, insufficiently active; and those with a score of 150 or more, sufficiently active to achieve health benefits [11].

### Physical activity barriers (PAB) questionnaire

The questionnaire on barriers to physical activity consisted of 24 items, derived from several questionnaires from previous studies [23-25]. All questions were presented in both English language and Bahasa Malaysia. Face validity, content validity and a pre-test were conducted on the selected items. Based on the pre-test, the Cronbach’s alpha value for the questionnaire was 0.859. These items were categorised into three domains: personal, physical environment and social environment. The Cronbach’s alpha values for each domain were 0.707, 0.741 and 0.687, respectively. According to Bland and Altman [26], questionnaire content with an alpha value of 0.70 or more is regarded as satisfactory. The personal domain included 15 items, the physical environment domain included five items, and the social environment domain included four items (Table 1). Each item was scored on a Likert scale ranging from 1 to 5, indicating ‘strongly disagree’, ‘disagree’, ‘neutral’, ‘agree’ and ‘strongly agree’. All items were positive statements, which meant that the higher the score, the higher the likelihood that the item was a barrier.

**Table 1:** Barrier items for each domain and percentage of subjects who agreed to the statements

**Item**	**Percentage (%)**
**Personal**	
I don’t have extra energy to do physical activity after finishing my work.	21.6
I feel sick and uncomfortable physically while exercising	13.2
I have health problems which prevent me from being physically active.	12.5
Physical activity is difficult and tiring.	12.7
I look funny and feel ashamed when doing physical activities.	6.7
I’m not interested in doing exercise or physical activities.	9.2
I don’t get pleasure from physical activities or exercise.	9.8
I think other recreational activities with friends or family members are more fun than exercise or physical activities.	38.6
I think physical activity is not beneficial to my health.	5.5
I’m afraid of injury and fear for my safety when exercising.	13.7
I’m too lazy to do physical activities.	18.3
Intensity of exercise required to get health benefits are too high for me.	21.6
I think I’m not talented in doing physical activities.	15.1
I’m lack of self-discipline/initiative in performing physical activities.	29.8
My body shape doesn’t allow me to do physical activities.	7.2
**Social Environment**	
My family members or friends don’t encourage me to do physical activities.	7.2
I don’t have friends to do physical activities together.	25.5
I don’t have free time to exercise or do physical activities because of my work.	27.7
I have to take care of my children or family members.	21.8
**Physical Environment**	
There are no facilities or places to do physical activities in my residential area.	19.8
Facilities or sports area are too far and I don’t have any transportation.	11.8
I don’t know how to use sports equipments or specialties in doing physical activities.	17.5
The hot weather or rainy days prevent me to do physical activities.	34.7
I don’t have extra money to go to the sports facilities such as gymnasium or to buy sports equipments and clothes.	27.5

Note: The percentage of each barrier was determined by calculating the percentage of subjects who had agreed to the statements (subjects who chose ‘agree’ and ‘strongly agree’).

### Statistical analyses

#### Grouping of subjects

The subjects were divided according to their age into three clusters, which were young (20–39 years), middle age (40–59 years), and elderly (60 years and above) clusters. Besides, subjects were also divided into subgroups according to their ethnicity, marital status, employment status, educational level, monthly household income (currency conversion: 1.00 USD = RM3.07), BMI, waist circumference, and physical activity score to assess the effects of such classification on the comparison of physical activity barriers mean values among subjects. The overall result was also presented. The normality of data was assessed using the Kolmogorov-Smirnov test.

### Percentage of the physical activity barriers

The percentage of each barrier was determined by calculating the percentage of subjects who had agreed to the statements (subjects who chose ‘agree’ and ‘strongly agree’).

### Comparison of mean between groups

The age, body anthropometric measurements, and physical activity score between the three age clusters were compared using one-way ANOVA if the distribution of the data was normal or Kruskal-Wallis test if the data were skewed. Total score of each domain of physical activity barriers was calculated by summing the score of each item in the domain and the total score of physical activity barriers was calculated by summing the total score of all three domains. The total score of each domain and total physical activity barriers score between Malay and Chinese subjects, single and married subjects, subjects who were working and unemployed, and subjects with waist circumference <90 and ≥90 cm were analyzed using independent t-test. One-way ANOVA with post hoc (Tukey) analysis was used to analyze the total score of each domain and total physical activity barriers score among different educational level, monthly household income, BMI, and physical activity status subgroups.

### Regression analysis

Multiple stepwise linear regression analysis was used to determine the influence of each factor on physical activity barriers. Factors involved were age, ethnicity (0 = Malay, 1 = Chinese), marriage status (0 = single, 1 = married), educational level (0 = primary and secondary education, 1 = college/university), monthly household income (0 = < RM3500, 1 = ≥ RM3500), physical activity status (0 = not active/sedentary, 1 = active), BMI and waist circumference. Beta-coefficient (β) was used to describe to describe the extent of SD change in the physical activity barriers score if the predictor of interest changed by 1 SD (while the other predictors were held constant). R^2^ was used to describe the variation of barriers score. The level of significance was set at p < 0.05. Data analysis was conducted using the Statistical Package for Social Sciences version 17.0 software (SPSS Inc., Chicago, USA).

## Results

### Sociodemographic characteristics

A total of 840 subjects were recruited during the screening sessions. One hundred five subjects were excluded because they did not complete the anthropometric measurements, physical activity questionnaire and physical activity barriers questionnaire. Data from 730 subjects (86.9% of the subjects from the original recruitment) consisting of 308 Malay men (42.2%) and 422 Chinese men (57.8%) were included for analysis. Sociodemographic characteristics of the subjects were presented in Table 2. The age of the subjects ranged from 20 to 83 years (mean = 47.1 years, SD = 14.5 years).

**Table 2:** **Socio**-**demographic characteristics of participants** (**n** = **730**)

**Characteristic**	**n (%)**
**Age group**	
Young adults (20–39 years old)	197 (27.0)
Middle aged adults (40–59 years old)	388 (53.1)
Elderly (≥60 years old)	145 (19.9)
**Ethnicity**	
Malay	308 (42.2)
Chinese	422 (57.8)
**Marital status**	
Married	579 (79.3)
Not married	151 (20.7)
**Educational level**	
Primary education	103 (14.1)
Secondary education	380 (52.1)
College/university	247 (33.8)
**Employment status**	
Employed	502 (68.8)
Unemployed	228 (31.2)
Monthly household income*	
Low (<RM1500)	195 (26.7)
Moderate (RM1500-RM3500)	290 (39.7)
High (>RM3500)	245 (33.6)

* Classification from Department of Statistics Malaysia (currency conversion: 1.00 USD = RM3.07).

The subjects were divided according to their age groups namely, young (n = 197), middle aged (n = 388), and elderly (n = 145) adults. The mean age of each group was 27.7 years (SD = 6.2 years) for young subjects, 50.0 years (SD = 5.8 years) for middle-aged subjects, and 65.4 years (SD = 6.5 years) for elderly subjects (Table 3). Descriptive analyses showed that 79.3% of subjects were married, 52.1% had secondary educational level, 68.8% were still working, and 39.7% had household income between RM1500 to RM3500 (based on Department of Statistics Malaysia) (Table 2).

**Table 3:** **The characteristics of the subjects according to age groups and as a whole** (**n** = **730**)

	**Age group**								**Significance**
	**Young (n** **=** **197)**		**Middle age (n** **=** **388)**		**Elderly (n** **=** **145**)		**Overall (n** **=** **730)**		
	**Mean**	**SD**	**Mean**	**SD**	**Mean**	**SD**	**Mean**	**SD**	
Age (years)	27.7	6.2	50.0	5.8	65.4	6.5	47.1	14.5	*, **, ***
Weight (kg)	69.2	14.8	71.4	11.7	67.2	11.5	70.0	12.7	***
Height (cm)	169.5	6.0	167.1	6.0	164.7	6.9	167.3	6.4	*, **, ***
BMI (kg/m^2^)	24.0	4.5	25.5	3.9	24.8	4.0	25.0	4.1	*
Waist circumference (cm)	86.0	10.5	90.7	9.9	90.2	10.3	89.3	10.4	*, **
	Median	IQR	Median	IQR	Median	IQR	Median	IQR	
Physical activity score (minutes)	120.0	178.0	87.5	180.0	90.0	149.0	90.0	180.0	

Physical activity score is presented in median and interquartile range (IQR) because it is skewed.

*p < 0.05 (significant difference between young and middle-aged men); **p < 0.05 (significant difference between young and elderly men); ***p < 0.05 (significant difference between elderly and middle-aged men).

### BMI, waist circumference, and physical activity

One-way ANOVA indicated significant difference (p < 0.05) in the weight, height, BMI, and waist circumference values among the three age clusters (Table 3). Elderly subjects were significantly shorter than the subjects in the clusters younger than them (p < 0.05). The young subjects had a significant lower BMI and waist circumference compared to both the middle-aged and elderly subjects (p < 0.05). Most of the subjects had a normal body weight (49.6%), while 34.8% of the subjects were overweight, 11.8% were obese and 3.8% were underweight. Meanwhile, 48.6% of the subjects were centrally obese (waist circumference more than 90cm). Most of the subjects (53.2%) presented a level of physical activity below 150 minutes on the 7 days before screening session (insufficiently active), 37.2% were active, whereas only 9.6% of the subjects scored 0 minutes per week (sedentary).

### Physical activity barriers

Table 1 shows the prevalence of each perceived barrier by all subjects. The perception that other recreational activities with family and friends are more fun was the most frequently reported barrier, followed by weather, lack of discipline, lack of free time, lack of money, and lack of friends (all these with a prevalence greater than 25%). The perception that physical activity is not beneficial to health and feeling ashamed were the least frequently reported barriers (5.5% and 6.7% respectively).

### The effects of sociodemographic characteristics on physical activity barriers

Subjects were categorized into subgroups according to their age, ethnicity, marital status, employment status, educational level, and monthly household income (Table 4). No significant difference could be observed in the physical activity barriers score among the three age clusters. Independent t-test indicated that Malay subjects had significantly higher personal barriers score (p < 0.001) and total physical activity barriers score (p = 0.007) than Chinese subjects. Married subjects and subjects who were still working had significantly higher social barriers score than single subjects (p = 0.002) and unemployed subjects (<0.001). One-way ANOVA showed significant differences in barriers score among subjects with distinct educational levels and monthly household income. Post hoc analysis indicated that subjects with primary educational level and subjects with household income less than RM1500 had significantly higher personal, physical environment and total physical activity barriers score than subjects with higher educational level and subjects with higher monthly household income (p < 0.001).

**Table 4:** Mean total scores of three main domains of physical activity barriers (n = 730)

**Variable/domain**	**Personal**	**Social environment**	**Physical environment**	**Total score**
	**Mean** ± **SD**	**Mean** ± **SD**	**Mean** ± **SD**	**Mean** ± **SD**
**Age cluster**				
Young	31.9 ± 8.3	8.7 ± 2.9	11.5 ± 3.6	52.2 ± 12.5
Middle age	32.5 ± 9.0	9.3 ± 2.8	12.0 ± 3.8	53.7 ± 13.2
elderly	32.8 ± 10.3	8.9 ± 2.8	12.1 ± 4.1	53.8 ± 15.1
*p* value	0.667	0.059	0.294	0.417
**Ethnicity**				
Malay	34.1 ± 9.0^a^	8.9 ± 2.7	11.8 ± 3.7	54.9 ± 13.3^a^
Chinese	31.2 ± 8.9	9.1 ± 3.0	11.9 ± 3.8	52.1 ± 13.4
*p* value	<0.001*	0.350	0.689	0.007*
**Marital status**				
Single	32.2 ± 7.7	8.4 ± 2.5	11.7 ± 3.5	52.2 ± 11.2
Married	32.4 ± 9.4	9.2 ± 2.9^a^	11.9 ± 3.9	53.6 ± 13.9
*p* value	0.717	0.002*	0.552	0.278
**Employment status**				
Unemployed	32.3 ± 9.4	8.3 ± 2.6	12.2 ± 3.9	52.8 ± 13.8
Employed	32.5 ± 8.9	9.4 ± 2.9^a^	11.7 ± 3.7	53.5 ± 13.2
*p* value	0.826	<0.001*	0.170	0.473
**Educational level**				
Primary	35.3 ± 9.4^a^	9.4 ± 3.1	13.7 ± 4.1^a^	58.5 ± 13.9^a^
Secondary	33.0 ± 9.1^a^	9.1 ± 2.9	12.0 ± 3.7^b^	54.0 ± 13.4^b^
Tertiary	30.3 ± 8.5^b^	8.8 ± 2.7	11.0 ± 3.4^c^	50.0 ± 12.3^c^
*p* value	<0.001*	0.166	<0.001*	<0.001*
**Monthly household income**				
<RM1500	34.7 ± 9.8^a^	8.9 ± 2.7	12.8 ± 4.0^a^	56.4 ± 14.2^a^
RM1500-RM3500	32.5 ± 8.6^b^	9.2 ± 3.0	12.0 ± 3.8^a^	53.6 ±13.0^a^
>RM3500	30.5 ± 8.6^c^	8.9 ± 2.8	11.0 ± 3.4^b^	50.7 ± 12.7^b^
*p* value	<0.001*	0.399	<0.001*	<0.001*
**BMI**				
Underweight	32.4 ± 10.0	9.0 ± 3.1	13.4 ± 3.8	54.8 ± 14.9
Normal	31.0 ± 9.0^a^	8.8 ± 2.9^a^	11.8 ± 3.9	51.5 ± 13.5^a^
Overweight	33.1 ± 8.7^b^	9.1 ± 2.8	11.6 ± 3.4	53.9 ± 12.8^a^
Obese	36.3 ± 8.8^c^	9.8 ± 2.7^b^	12.4 ± 4.2	58.5 ± 13.3^b^
*p* value	<0.001*	0.034*	0.073	<0.001*
**Waist circumference**				
<90cm	31.2 ± 8.9	8.8 ± 3.0	12.0 ± 3.8	51.9 ± 13.4
≥90cm	33.7 ± 9.1^a^	9.3 ± 2.8^a^	11.8 ± 3.7	54.8 ± 13.3^a^
*p* value	<0.001*	0.043*	0.546	0.003*
**Physical activity**				
Sedentary	38.2 ± 7.5^a^	10.7 ± 2.4^a^	13.4 ± 3.3^a^	62.4 ± 10.1^a^
Insufficiently active	32.5 ± 8.9^b^	9.1 ± 2.8^b^	12.0 ± 3.7^b^	53.5 ± 13.0^b^
Sufficiently active	30.8 ± 9.0^c^	8.5 ± 2.8^c^	11.4 ± 3.9^b^	50.7 ± 13.8^c^
*p* value	<0.001*	<0.001*	<0.001*	<0.001*

Note: Independent t-test and one-way ANOVA were used to compare the mean of total scores of each domain. Different alphabets on the same column shows the significant difference between groups using Tukey test.

*significant difference at p < 0.05.

### The effects of BMI and waist circumference on physical activity barriers

Subjects were categorized into subgroups according to their BMI and waist circumference (Table 4). One-way ANOVA and post hoc analysis indicated that obese subjects had higher personal (p < 0.001), social environment (p = 0.034), and total physical activity barrier score (<0.001) than overweight and normal subjects. On the other hand, independent t-test indicated similar results for waist circumference comparison, in which subjects with waist circumference more than 90 cm also had higher personal (<0.001), social environment (p = 0.043) and total physical activity barrier score (p = 0.003) than subjects with waist circumference less than 90 cm.

### The effects of physical activity on physical activity barriers

Subjects were categorized into subgroups according to their physical activity status (Table 4). One-way ANOVA and post hoc analysis revealed that sedentary subjects had significantly higher personal, social environment, physical environment, and total physical activity barrier score than insufficiently active subjects and also active subjects (p < 0.001).

### Regression analysis

Stepwise linear regression analysis was conducted for each domain of physical activity barriers (Table 5). For personal barriers, marriage status (β = −0.101, p < 0.001), BMI (β = 0.180, p < 0.001), and physical activity status (β = −0.215, p < 0.001) were selected as the significant predictors of the barriers. They described 8.7% of the variation in personal barriers score. BMI exerted positive effect on personal barriers score, while being married and sufficiently active exhibited negative effects on the barriers score. For social environment barriers, BMI (β = 0.112, p = 0.024) and physical activity status (β = 0.152, p = 0.002) were selected as significant predictors of the barriers. They described 3.7% of the variation in social environment barriers score. BMI exerted positive effect on social environment barriers score while being sufficiently active had negative effect on the barriers score. For physical environment barriers, educational level (β = −0.132, p = 0.011), monthly household income (β = −0.112, p = 0.031), and physical activity status (β = −0.130, p = 0.008) were selected as significant predictors of the barriers. They described 6.1% of the variation in physical environment barriers score. Being highly educated, higher monthly household income and sufficiently active exhibited negative effects on physical environment barriers score. When analyzed as a whole, BMI (β = 0.146, p = 0.003), educational level (β = −0.110, p = 0.023), and physical activity status (β = −0.210, p < 0.001) were selected as significant predictors of the total barriers score. BMI exerted positive effect on total barriers score while being highly educated and sufficiently active had negative effects on the score. Overall, these factors described 8.5% of the variation in total barriers score (Table 5). The equation for each model was listed in Table 6.

**Table 5:** Multiple regression results of the subjects according to barrier domains and as a whole (n = 730)

**Barrier domain**	**Predictor (s)**	**β coefficient**	**p value (predictor)**	**R**^**2 **^**(model)**	**p value (model)**
Personal	Physical activity status	−0.215	<0.001	0.087	<0.001
	BMI	0.180	<0.001		
	Marriage status	−0.101	<0.001		
Social environment	Physical activity status	−0.152	0.002	0.037	0.001
	BMI	0.112	0.024		
Physical environment	Educational level	−0.132	0.011	0.061	<0.001
	Physical activity status	−0.130	0.008		
	Monthly household income	−0.112	0.031		
Overall	Physical activity status	−0.210	<0.001	0.085	<0.001
	BMI	0.146	0.003		
	Educational level	−0.110	0.023		

Note: Beta-coefficient (β) was used to describe to describe the extent of SD change in the physical activity barriers score if the predictor of interest changed by 1 SD (while the other predictors were held constant). R^2^ was used to describe the variation of barriers score

*significance level at p < 0.05.

**Table 6:** Equations of the regression analysis according to the model studied

**Model**	**Equation**
Personal	Barriers score = 33.076 – 4.260 (physical activity) + 3.279 (BMI) – 2.518 (marriage status)
Social environment	Barriers score = 9.134 – 1.003 (physical activity) + 0.674 (BMI)
Physical environment	Barriers score = 12.867 – 1.060 (educational level) – 1.100 (physical activity) – 0.888 (household income)
Overall	Barriers score = 53.281 – 6.237(physical activity) + 3.984 (BMI) – 3.119 (educational level)

## Discussion

This study determined the barriers to physical activity perceived by Malaysian men and the relationship between these barriers with sociodemographic backgrounds, BMI, waist circumference, and physical activity status. The findings of this study are in agreement with other studies that have evaluated physical activity barriers in Japan, the United States and Brazil [9-11]. However, this study provided insight into the effects of various personal, social and physical environment factors on perceived physical activity barriers among men, which in turn influenced the physical activity level. Marriage status, educational level, household income, BMI, and physical activity status were proven to be associated with perceived barriers.

Higher BMI and being single were associated with having more personal perceived barriers. Previous research has shown that being overweight may be a significant barrier to physical activity [27], which may be related to the misperception that these men are incapable of engaging in a healthy lifestyle because they are overweight or obese [28] Perception of being overweight has been suggested as a cognitive barrier to physical activity [28]. Furthermore, individuals with high BMI have reported fear of injuries and having an injury or disease as barriers to physical activity [11]. Lack of confidence and motivation among overweight men may also prevent them from being physically active.

Marriage status has been associated with physical activity. King et al. [29] found that the transition from being single to married gave a positive effect to physical activity compared to individuals who stayed single. The present of the other half might influences and motivates the individual to engage in physical activity. Furthermore, motivation factor was proved to have relationship with physical activity level [12]. Interventions and health-promotion programmes using a cognitive approach seem most suitable for overcoming these barriers. For example, strategies to increase confidence and motivation may help individuals to increase physical activity participation and improve weight management.

Social support from family and friends is important to encourage participation in physical activity. Several studies have reported the lack of support from family members, no friends to do physical activities together and no role model as guidance as social barriers among adults [9,30,31] which are similar with this study. Participation in physical activity with other people can help in developing positive social norm for physical activity in the individual’s social network [32]. Observing the physical activity behaviour of others can also help individuals learn about physical activity, in addition to receiving the positive feedback about the benefits of physical activity [33].

Monthly household income was found to be associated with having more physical environment barriers. Furthermore, lack of money to buy sports equipments and to go to sports facilities was also one of the most frequent reported barriers, as noted in the previous study in Brazil [11]. This might be due to a perception that the health benefits of physical activity can only be attained by going to a gym or playing certain sports, which may be expensive. The cheapest and effective way to be more physically active is by walking and it can be the alternative for people who have financial problems. However, most neighbourhoods in Malaysia do not have accessible recreation facilities, such as walking and cycling paths, to encourage physical activity in the community [6]. Physical environment can influence physical activity behaviour by either promoting or discouraging physical activity through factors such as access to safe recreation, accessibility of recreation facilities and transit option [10]. Therefore, policy-level interventions and development regulations, such as provisions for parks and cycling paths, would help to overcome this problem. Furthermore, health-promoting programmes or intervention programmes should emphasise community education on physical activity to rectify the misperception about physical activity.

Educational level was also found to contribute significantly towards physical activity barriers. Men with higher educational level were more likely to perceive fewer barriers than men with lower educational level. These individuals who are usually at the highest levels of income and job classifications too are more likely to engage in healthy behaviours such as physical activity engagement and proper diet than those of lower job status and incomes [34]. They also tend to adopt more health-promoting behaviours and reduce riskier behaviours at a faster rate. This might be due to the high awareness of the benefits of living a healthy lifestyle and the capability to obtain social and material resources (such as gym memberships) that maintain physical activity even in adverse weather conditions [35].

On the other hand, individuals with lower educational level might be dealing with stressful environments because of work and poor lifestyle that can influence the uptake of physical activity [35]. However, this result is different from the Japanese study [9], whereby, highly educated Japanese men perceived more barriers compared to less educated men. This suggests that the associations between types of barriers and population characteristics vary according to cultural background.

### Strengths and limitations

This study relied on a population-based data collection and included adults aged 20 years and above from different sociodemographic backgrounds. All questionnaires and equipments had been validated and the enumerators had been trained and supervised to obtain high-quality information. The inclusion of a wide age range and different physical activity status was important to provide an insight of the association between physical inactivity and barriers. Three different domains of physical activity barriers were also included in this study which is useful for the exploration of various barriers involved in performing physical activity. This study was also believed to be the first population-based study in Malaysia to investigate perceived barriers in physical activity.

Several limitations in the present study need to be considered in the interpretation of the results presented. The subjects were recruited via purposive sampling, nonrandomized rather than a randomized sampling; hence selection biases might prevent generalization of the findings to the population. This study was a part of larger health study and interview length was a concern, only IPAQ short-form could be used to assess physical activity. The questionnaire does not discriminate leisure-time physical activities to other domains (occupation, commuting and housework). Previous studies found that voluntary or leisure-time physical activities were associated with perceived barriers [11]. In the present study, the discrimination between leisure-time physical activities and other domains (occupation, commuting and housework) was not made, so we could not find out which type of activity exerted greater influence on the barriers score of the subjects.

## Conclusions

Marriage status, educational level, household income, BMI, and physical activity status were proven to be associated with perceived barriers. The perception that other recreational activities with family and friends are more fun was the most frequently reported barrier, followed by weather, lack of discipline, lack of free time, lack of money, and lack of friends. To increase participation in physical activity, policy makers should consider the significant barriers (personal, social and environmental) when developing appropriate intervention programmes. Health-promoting strategies to increase awareness, knowledge, skills and motivation related to physical activity are required to encourage men to be physically active. Additional strategies to promote healthy physical activity among overweight men may need to also focus on weight status barriers.

## Competing interest

The authors declare that they have no competing interest.

## Authors’ contributions

SI was involved in data collection, analysis and interpretation of data as well as drafting the manuscript. NAK has given critical input in data interpretation and manuscript development. NLO and WZWN had revised the manuscript critically for important intellectual content. All authors had read and gave final approval of the version to be published.

## Pre-publication history

The pre-publication history for this paper can be accessed here:

http://www.biomedcentral.com/1471-2458/13/275/prepub
